# Emotional Welfare and Its Relationship with Social Interactions and Physical Conditions of Finishing Pigs in Lairage at the Slaughterhouse

**DOI:** 10.3390/ani15081108

**Published:** 2025-04-11

**Authors:** Alexandra Mendes, C. Saraiva, J. G. Díez, M. Almeida, F. Silva, I. Pires, Sónia Saraiva

**Affiliations:** 1 CECAV—Veterinary and Animal Research Centre, University of Trás-os-Montes and Alto Douro, 5000-801 Vila Real, Portugal; alexxana27@gmail.com (A.M.); crisarai@utad.pt (C.S.); juangarciadiez@utad.pt (J.G.D.); mdantas@utad.pt (M.A.); fsilva@utad.pt (F.S.); ipires@utad.pt (I.P.); 2 Department of Veterinary Sciences, School of Agricultural and Veterinary Sciences, University of Trás-os-Montes e Alto Douro (UTAD), 5000-801 Vila Real, Portugal; 3 Associate Laboratory for Animal and Veterinary Science (AL4AnimalS), 1300-477 Lisboa, Portugal

**Keywords:** descriptor, qualitative behavior assessment (QBA), holding pen, frustration

## Abstract

This study was conducted in a Portuguese slaughterhouse for finishing pigs. A total of 93 batches with an average slaughter weight of 93.3 kg (±0.6) were assessed in the lairage pens using the qualitative behavior assessment (QBA) tool to evaluate both positive and negative descriptors. Social interaction behaviors and the physical conditions of pigs in each batch were also measured. Physical examination revealed that pigs with body condition scores of 4 or 5 were less active and playful, presenting negative correlations with these descriptors. Groups of pigs at rest showed negative correlations (*p* < 0.001) with active (r = −0.338), lively (r = −0.352), and playful (r = −0.424) behaviors, while resting behavior was positively correlated (*p* < 0.01) with relaxed groups (r = 0.331). The presence of fighting and mounting the backs of other animals was highly correlated (*p* < 0.001) with negative emotional states like agitation, distress, irritability, and tense. Batches exhibiting positive emotional states, such as calmness and relaxation, showed significant associations with behaviors like fighting and mounting the backs of other animals. This indicates that experiencing positive emotions can lead to a decrease in aggressive or stress-related behaviors. Ear biting correlated with the descriptor “bored”, indicating a potential relationship between this behavior and a lack of environmental stimulation. The descriptors “frustration” and “bored” (*p* < 0.001) showed an association with the behavior of chewing without a substrate, regardless of whether fights occurred. Descriptors like ”active”, ”friendly”, “positively occupied”, ”sociable”, and “lively” have higher loadings on principal component 1 (PC1), reflecting positive affective welfare. In PC2, descriptors representing relaxation are positioned as opposite to terms like “irritable”, “distressed”, and “tense”, suggesting that PC2 could be useful for identifying conditions of high stress in pigs. These findings underscore the relationship between emotional states and behaviors, suggesting that combining QBA with behavioral observations provides a comprehensive framework for assessing animal welfare. Improving pig welfare could be achieved by reducing aggression and enhancing environmental enrichment to alleviate boredom during lairage.

## Simple Summary

Good animal welfare goes beyond merely preventing negative experiences; it also involves recognizing and promoting positive emotional states. Qualitative behavior assessment (QBA) is particularly valuable in this context, as it allows for the identification of such positive states by evaluating how animals interact with their surroundings. QBA, therefore, can contribute to welfare assessment because it can capture variation in how animals respond to and deal with their environment in that instant. In this study, 93 transport batches of 19,582 animals were evaluated in lairage pens using the QBA tool to assess both positive and negative welfare descriptors. The findings highlight a strong relationship between emotional states and specific behaviors in groups of pigs, such as fighting or mounting behaviors. The groups of pigs that were more ”calm” and ”relaxed” displayed lower levels of aggressive behavior. Strategies aimed at reducing boredom and frustration are essential. These results reinforce the need for environmental enrichment and social engagement in lairage pens, as these factors contribute to a more stable and well-balanced environment, ultimately enhancing overall animal welfare.

## 1. Introduction

Slaughterhouses are a valuable source of information for monitoring animal health and welfare [1]. Animal-based indicators collected from pigs in holding pens or carcasses are commonly applied to assess management practices and pig welfare on-farm and during the pre-slaughter phase [2]. However, the monitoring of pig behavior has been limited. Pigs, both on farms and during transport, face several stress factors that can compromise their welfare [3]. These include factors such as food deprivation, exposure to loud noises, temperatures outside their comfort zone, unfamiliar environmental conditions, high stocking density, prolonged time spent in lairage, mixing with unfamiliar animals, and recent mixing of different genders [4,5,6]. Lairage can be often a source of stress since animals will be placed in a novel environment where social stress, fighting, and disturbed rest should be minimized [7,8]. Large group sizes with more unfamiliar pigs may prolong fighting during lairage time [9]. Also, sound intensity higher than 85 dB can be stressful for pigs [7]. The presence of these stress factors on the day of slaughter can negatively impact meat quality, leading to the development of pale, soft, and exudative (PSE) meats [8,10]. In addition, aspects such as the farm of origin or the genetic characteristics of the pigs may influence the response to different stressful situations [8,11]. Social interactions among pigs can be classified as social, aggressive, or exploratory. These interactions provide valuable information regarding their welfare, social dynamics, and environmental influences. However, the impact of social interactions on animals’ emotional experiences remains poorly understood [12]. Pigs have distinct needs related to their natural behaviors and physical health, which are crucial for ensuring their overall welfare [13]. Positive social interactions, such as rooting, exploring, and socializing, are very valuable indicators of the animal’s emotional state [14]. The effects of aggression among pigs have been well documented in terms of injuries and physiological responses; however, the emotional experience of animals in such contexts remains poorly understood [12]. Qualitative behavior assessment (QBA) is a welfare evaluation tool that employs a holistic approach to assess and capture an animal’s emotional state [15]. In the past, animal welfare research primarily focused on studying negative effects [16]. Today, good welfare is recognized as a complex, multifaceted concept that extends beyond the mere absence of negative experiences, emphasizing the increasing need for methods to identify and promote positive emotional states in animals [15,17]. QBA has proven to be an effective method for detecting positive effects in pig farms [12,18,19]. However, QBA can be applied under a range of conditions and can identify subtle differences in qualitative behavioral expression that can be important for welfare assessment and may otherwise be overlooked when individual behaviors are quantified [20]. QBA is a simple and versatile tool suitable for in situ assessments, capturing a wide range of expressive behaviors that reflect the animal’s emotional and physiological state [17,20]. This contrasts with physiological markers, which often focus on specific stress indicators like cortisol levels or heart rate and often involve blood draws or sensors requiring laboratory analysis and time for processing.

QBA is non-invasive and does not require physical contact or restraint, reducing the risk of additional stress [12]. However, research on the emotional state of pigs during lairage at the slaughterhouse is limited, and comprehensive research specifically focusing on emotional welfare during lairage time remains scarce. This experiment aimed to apply QBA to different batches of finishing pigs in holding pens and relate their emotional status to social interaction behaviors and their physical conditions. The findings of this study could help implement targeted strategies to enhance animal welfare, guide industry practices, and provide valuable insights to welfare authorities on advancements in animal welfare.

## 2. Materials and Methods

This study was carried out in a slaughterhouse for finishing pigs in Portugal from March to April (corresponding to the end of winter and the beginning of spring). A total of 93 batches were evaluated, with an average transport dimension of 210 pigs (±3.06), ranging from 60 to 241 pigs per batch. This evaluation comprised a total of 19,582 animals. The average weight of the batches was 93.3 kg (±0.6), with a range from 80 to 100 kg. The batches were of mixed gender and consisted of docked pigs for indoor intense production. The batches were selected randomly each day. On the majority of the days, the data were collected from 3 different batches, and on some days, the data were collected from 2 different batches. The batches of animals at the lairage were divided into holding pens according to their maximum capacity, not surpassing 235 kg/m^2^ (there were 3 holding pens with a maximum capacity of 27 animals, 27 holding pens with a maximum capacity of 30 animals, 12 holding pens with a maximum capacity of 45 animals, and 16 holding pens with a maximum capacity of 52 animals). The holding pens consisted of solid concrete floors with metal bars delimiting the pens (water was available but not food). The average temperature in the pens was 12.5 °C, ranging from a minimum of 2 °C to a maximum of 21 °C, with an average humidity of 78%. The data were obtained at the lairage pens, the collection of which involved conducting physical examinations of the animals, monitoring their social interactions, and performing QBA.

### 2.1. Qualitative Behavior Assessment (QBA)

In three different periods of the day (1st period—5:30 a.m. to 7:30 a.m.; 2nd period—7:30 a.m. to 9:30 a.m.; 3rd period—9:30 a.m. to 11:00 a.m.), the observatory was placed near one of the parts of the holding pens with animals from the batches already randomly selected to be evaluated. Firstly, the number of animals in the selected holding pen was counted and then the behavior of that group of animals was assessed using the QBA methodology [4,15]. The observer checked the correct visibility of the animals during the stipulated time in each period. The expressive quality of the animals’ activities was observed at the group level, focusing on their collective behaviors and interactions. At the observation point for each group of animals, the observation duration was at least 10 min and less than 20 min. Twenty given adjectives (descriptors) were used to evaluate the behavior per group, of which eleven were positive (active, relaxed, calm, content, friendly, positively occupied, lively, playful, sociable, inquisitive, and happy) and nine were negative (fearful, agitated, indifferent, frustrated, bored, irritable, tense, apathetic, and distressed) [4,15]. For each adjective, a visual analogue scale (VAS) of 125 mm was assigned. After the observation was completed, the 20 descriptors/adjectives were scored using the visual analogue scale (VAS). Based on the observation, a vertical mark was placed on the scale to indicate whether the descriptor was absent (0 mm) or dominant (125 mm) for the animals under study. The length (in mm) on the VAS was then measured with a ruler for each adjective. The lengths obtained indicated whether a certain adjective was of low prevalence (closer to 0 mm) or more prevalent (closer to 125 mm) in a group.

Table 1 below presents the definitions of the descriptors used in the QBA tool.

### 2.2. Training of the Observer

The observer was made familiar with the concept of QBA by reading relevant scientific literature. Detailed training sessions were conducted from nine video clips selected, each of 2 min duration and showing groups of pigs from different farms. The pigs were observed in groups (~20) to align the observer’s understanding of the assessment criteria, ensuring consistency and reproducibility.

### 2.3. Behavioral Assessment

Additional observed behaviors were recorded for the same group of animals during the same time period as the QBA (Table 2). These behaviors were categorized per group as either absent (score 0) or present (score 1).

### 2.4. Physical Examination

The physical evaluation of the pigs was performed by dividing the group previously observed in the behavioral examination into two parts. For each part of the holding pens, the observer recorded the total number of animals (used to verify if the capacity of the holding pen was surpassed or not, which it never was), the number of animals resting (lying down motionless), the number of animals panting (breathing rapidly in short gasps through the mouth), and the number of animals shivering (animals showing slow and irregular vibrations of any body part or the entire body) to evaluate resting and thermal comforts [4]. Additional observations included the body condition of the animals (scored from 1 to 5), the number of animals with manure on their body, and the percentage of the body covered with manure (visually assessed on one side). The number of animals coughing, the number of animals with rectal prolapses, the number of animals with visible hernias, and the number of animals showing signs of tail biting (e.g., tails with fresh blood, evidence of swelling, missing tissue, or a crust formation) were recorded to evaluate the degree of injuries and diseases [4]. The results were displayed as percentages.

### 2.5. Statistical Analysis

The relationships between the 20 descriptors and fighting, ear biting, tail biting, mounting the backs of other animals, and chewing without a substrate were analyzed using Spearman’s rank correlation coefficient, with statistical significance set at *p* < 0.01. The Kruskal–Wallis test was used to assess the differences in behavior (e.g., fighting, mounting, chewing without a substrate) across different emotional states as measured by QBA. Behaviors and physical conditions that did not achieve significant results (*p* < 0.05), such as tail or ear manipulations, were excluded from the reported findings. A principal component analysis (PCA) was applied to analyze results from QBA, including the descriptors “active”, “relaxed”, “fearful”, “agitated”, “calm”, “content”, “indifferent”, “frustrated”, “friendly”, “bored”, “playful”, “positively occupied”, “lively”, “inquisitive”, “irritable”, “tense”, “sociable”, “apathetic”, “happy”, and “distressed”. We reduced the number of variables, as some had no significant impact on the outcome. Variables were selected based on factor loading (FL) modules higher than 0.50 in absolute values and commonalities (CMs) higher than 0.7. Appropriateness was confirmed by Bartlett’s sphericity test (*p* < 0.0001). The number of components retained in the final evaluation was based on the Kaiser–Meyer–Olkin (KMO) criterion for the analysis of eigenvalues (>1) and the proportion of variance retained (>70%).

## 3. Results

Table 3 and Table 4 present the relationships evaluated between emotional states (positive and negative, respectively) and social interaction behaviors.

Batches with positive emotional states, such as “calm” and “relaxed”, were found to be negatively correlated with the occurrence of fights and mounting the backs of other pigs, indicating that these states are generally associated with a reduction in aggressive or stress-related behaviors. The ”playful” and “positively occupied” states displayed a relationship to certain behaviors (e.g., tail manipulation) (Table 3).

Both the presence of fights and mounting the backs of other animals were highly correlated (*p* < 0.001) with negative emotional states like agitation, distress, irritability, and unease. Negative states such as boredom and frustration show a positive correlation with the presence of other behaviors such as ear biting or chewing without a substrate (Table 4).

The Spearman correlations between the QBA descriptors and the results from the physical examination revealed that pigs with body condition scores of 4 or 5 exhibited significant negative correlations (*p* < 0.001) with the descriptors “active” and “playful”. Groups of pigs resting presented negative correlations (*p* < 0.001) with “active” (r = −0.338), “lively” (r = −0.352), and “playful” (r = −0.424). In contrast, resting behavior was positively correlated (*p* < 0.01) with “relaxed” groups (r = 0.331). Shivering also exhibited negative correlations (*p* < 0.01) with the descriptors “active” (r = −0.304), “lively” (r = −0.163), and “playful” (r = −0.307). Panting did not show many significant correlations with the QBA descriptors.

Table 5 presents the results of the Kruskal–Wallis test, showing the association between each evaluated emotional state from the QBA and specific social behaviors, including fighting, mounting another animal’s back, and chewing without a substrate.

Emotions such as agitation, tension, distress, and irritability were positively associated (*p* < 0.001) with mounting behavior. However, these emotions did not influence chewing behaviors (Table 5). Moreover, when fighting was present in a group (score 1), the group was more agitated, distressed, frustrated, irritable, and tense. In contrast, when fighting was absent (score 0), the group was generally calmer, more relaxed, content, and indifferent. Some positive emotional states (e.g., being calm, content, and relaxed) were negatively associated with the behavior of mounting the back of another animal. Additionally, groups that were bored or frustrated showed an increased tendency to engage in chewing without a substrate, while more playful and positively occupied states were associated with a decrease in this behavior.

**Figure 1:**
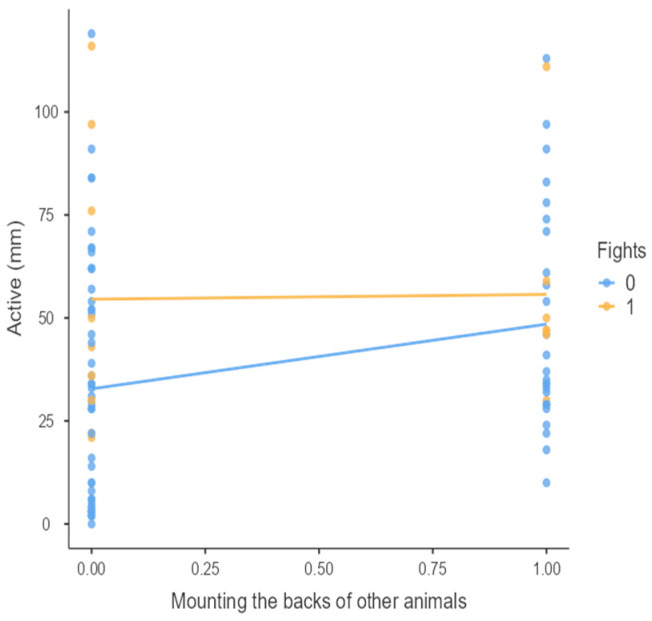
Mounting the backs of other animals vs. level of active behavior in groups (mm).

**Figure 2:**
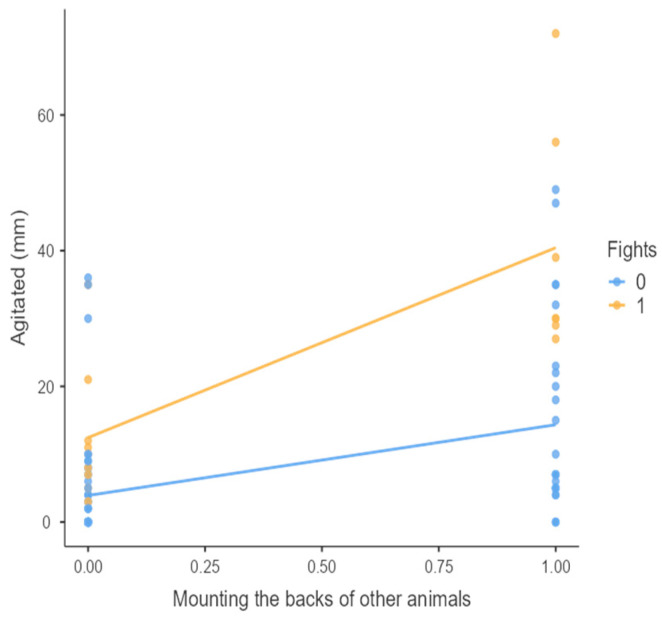
Mounting the backs of other animals vs. level of agitated behavior in groups (mm).

**Figure 3:**
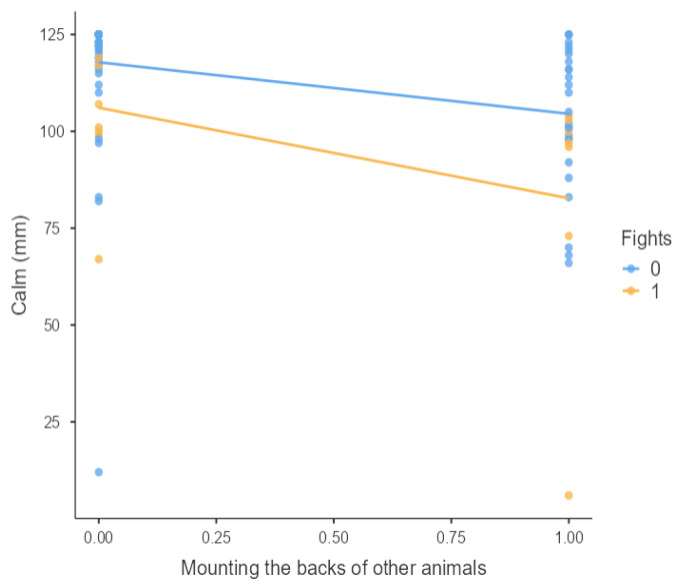
Mounting the backs of other animals vs. level of calm behavior in groups (mm).

**Figure 4:**
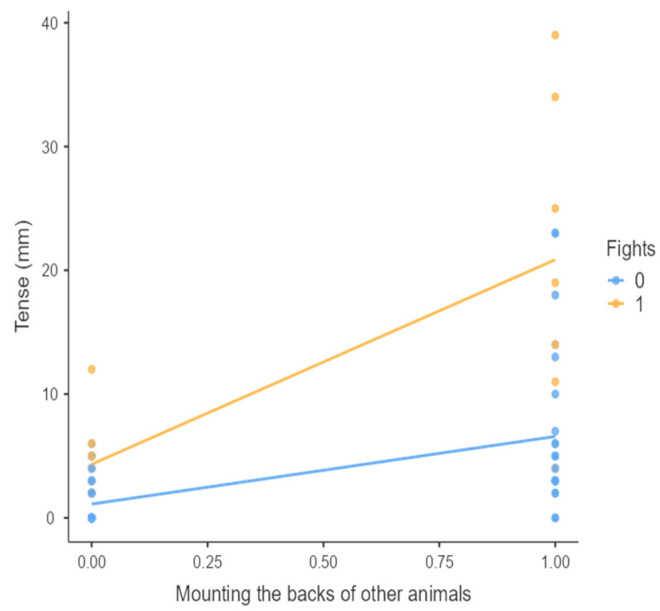
Mounting the backs of other animals vs. level of tense behavior in groups (mm).

**Figure 5:**
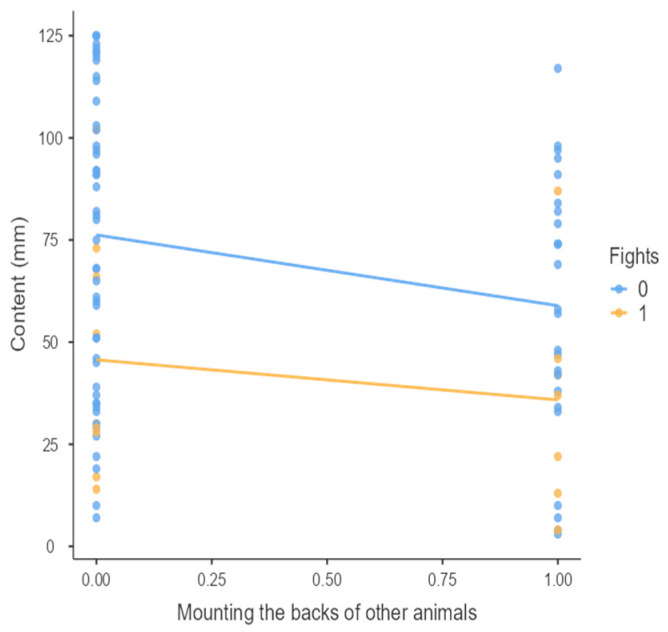
Mounting the backs of other animals vs. level of content behavior in groups (mm).

**Figure 6:**
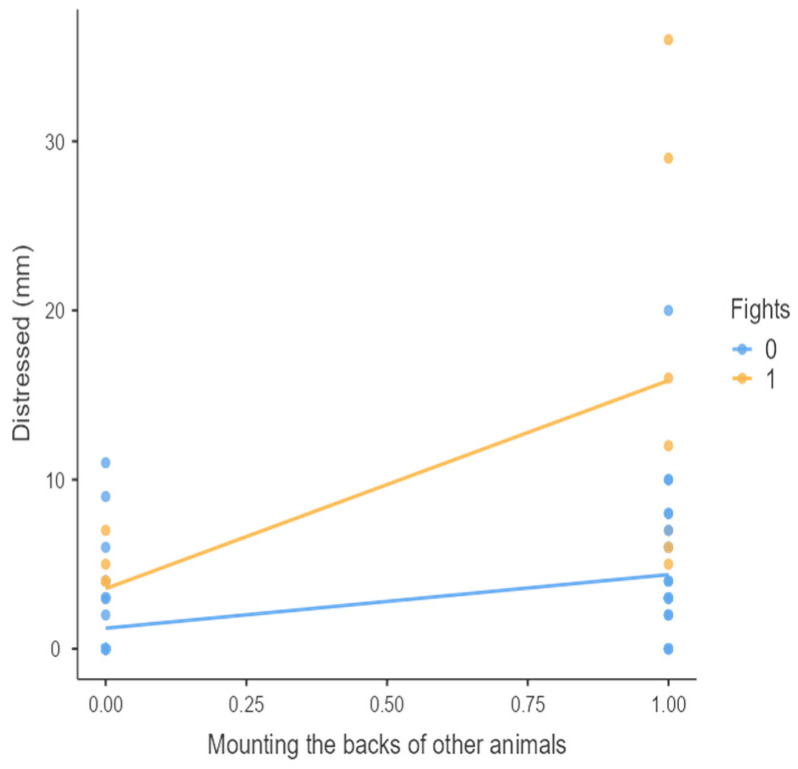
Mounting the backs of other animals vs. level of distressed behavior in groups (mm).

**Figure 7:**
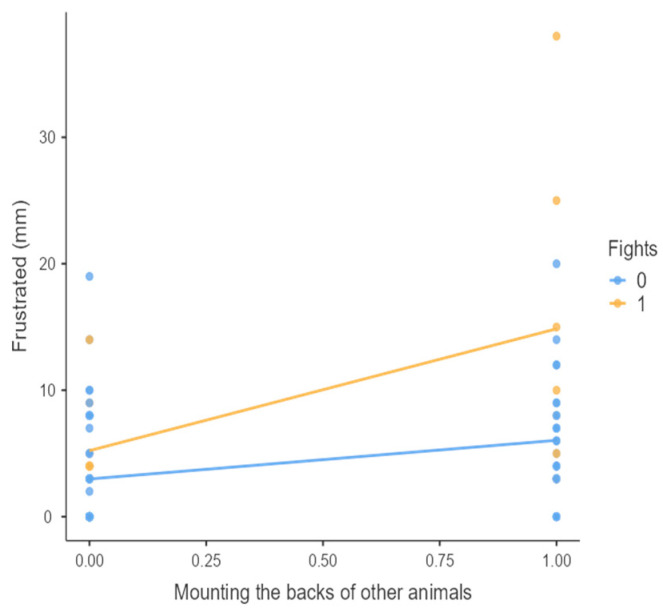
Mounting the backs of other animals vs. level of frustrated behavior in groups (mm).

**Figure 8:**
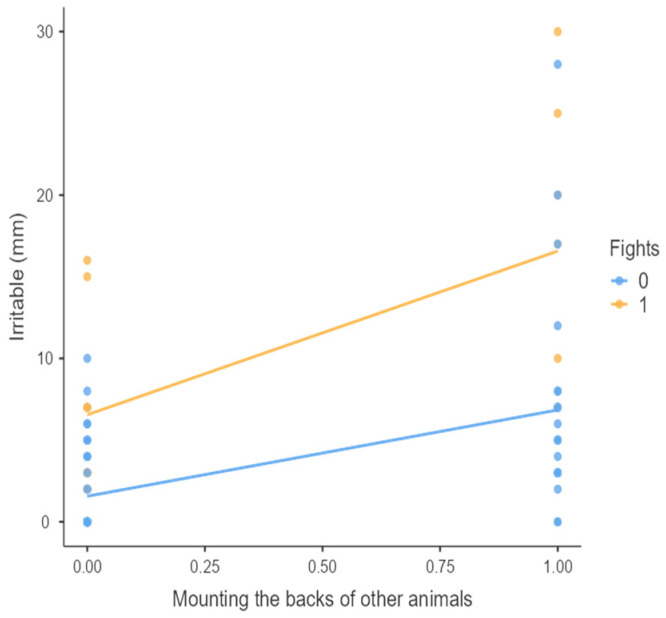
Mounting the backs of other animals vs. level of irritable behavior in groups (mm).

**Figure 9:**
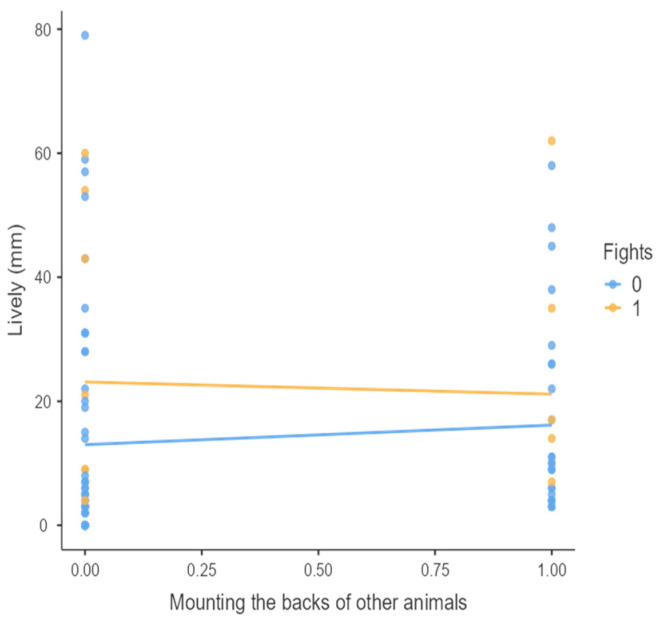
Mounting the backs of other animals vs. level of lively behavior in groups (mm).

**Figure 10:**
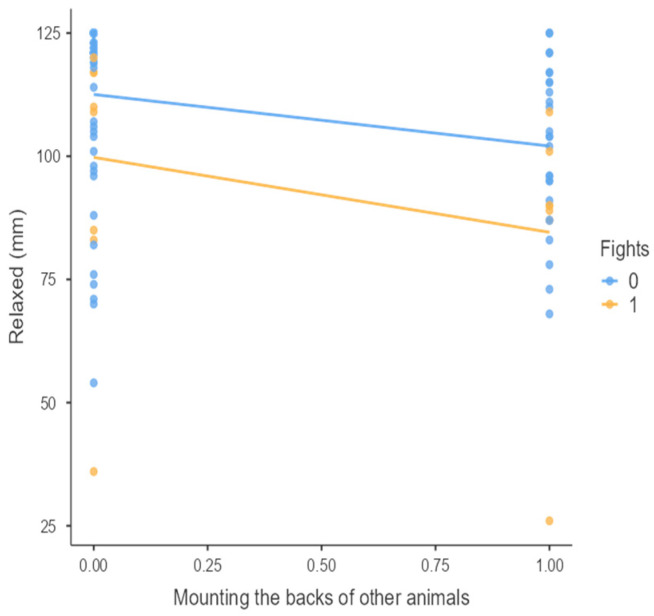
Mounting the backs of other animals vs. level of relaxed behavior in groups (mm).

**Figure 11:**
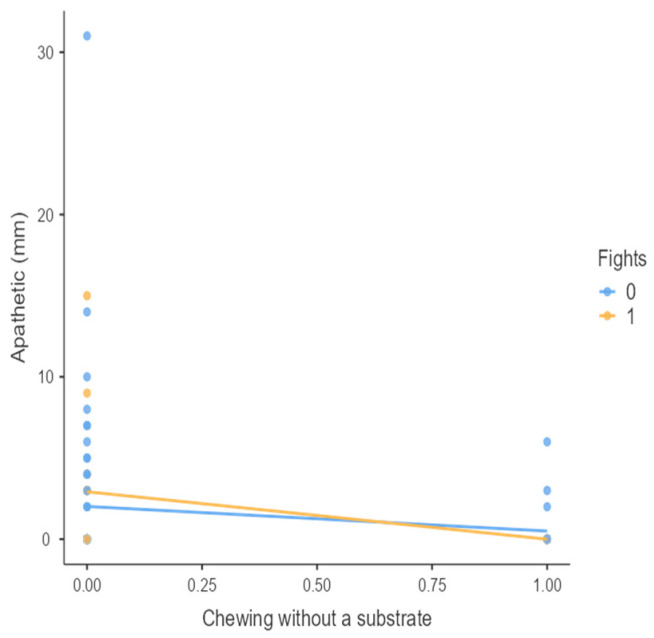
Chewing without a substrate vs. level of apathetic behavior in groups (mm).

**Figure 12:**
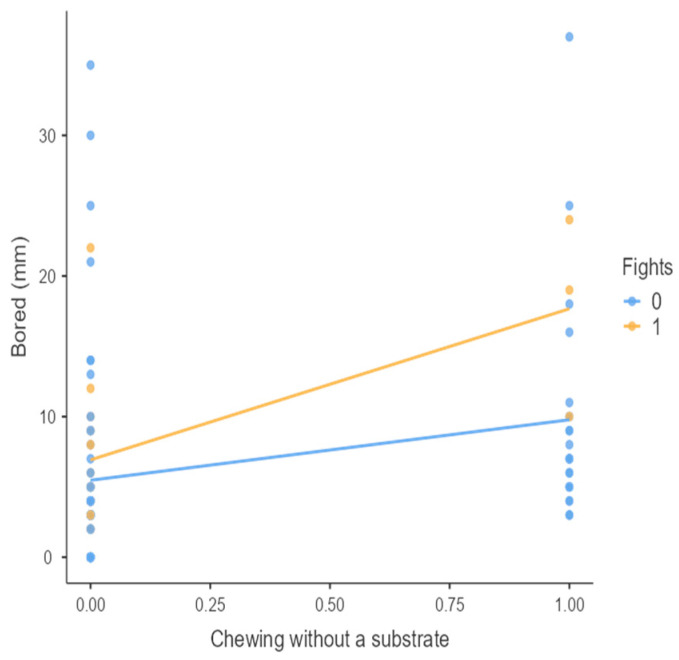
Chewing without a substrate vs. level of bored behavior in groups (mm).

**Figure 13:**
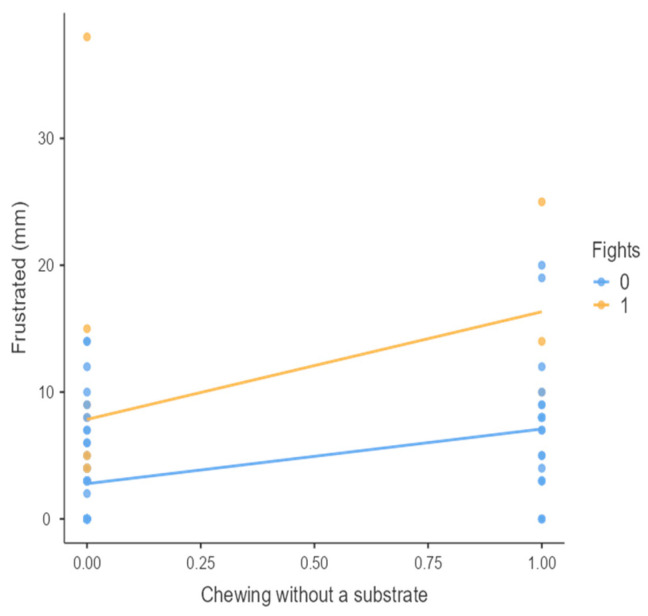
Chewing without a substrate vs. level of frustrated behavior in groups (mm).

**Figure 14:**
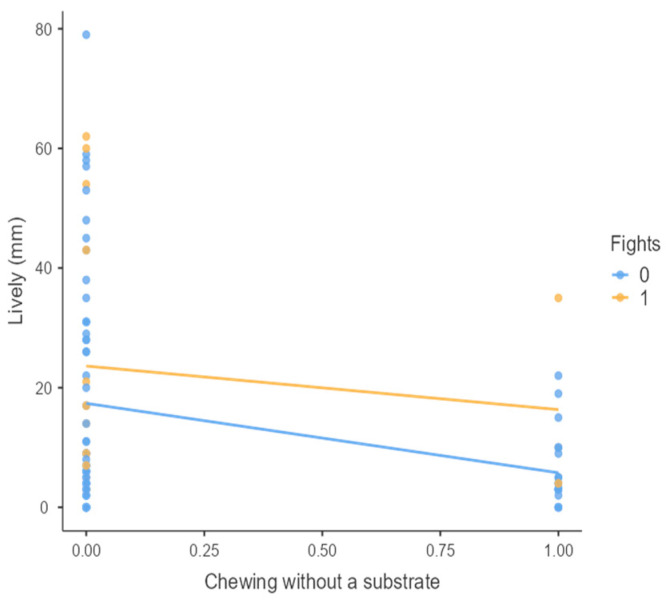
Chewing without a substrate vs. level of lively behavior in groups (mm).

**Figure 15:**
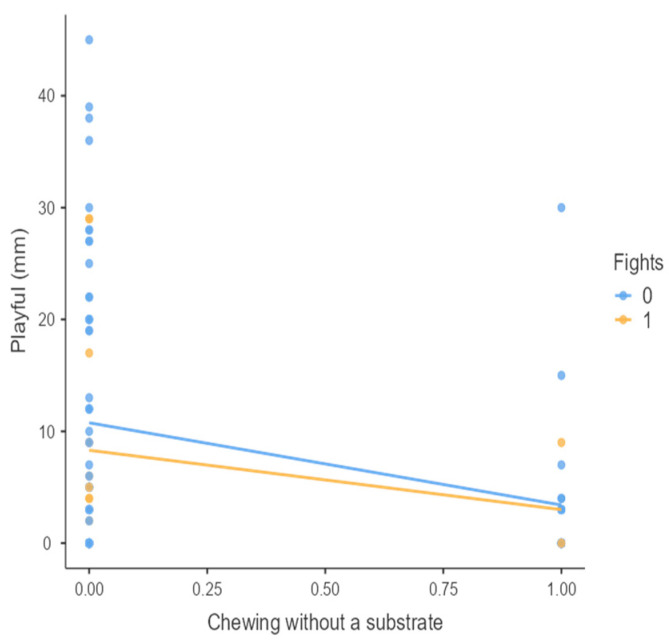
Chewing without a substrate vs. level of playful behavior in groups (mm).

The results from the non-parametric tests are represented in the figures below, visually showing the associations between emotional states from the QBA and specific behaviors (such as mounting the back of another animal, chewing without a substrate, and fighting). The relationship between fighting behavior (score 0 or 1), mounting the back of another animal (score 0 or 1), and chewing without a substrate (score 0 or 1) and the descriptors of the QBA is presented in Figure 1, Figure 2, Figure 3, Figure 4, Figure 5, Figure 6, Figure 7, Figure 8, Figure 9, Figure 10, Figure 11, Figure 12, Figure 13, Figure 14 and Figure 15.

For the descriptors “active” (Figure 1), “agitated” (Figure 2), “tense” (Figure 4), “distressed” (Figure 6), “frustrated” (Figure 7 and Figure 13), “irritable” (Figure 8), “bored” (Figure 12), and “lively” (Figure 14), the presence of fighting was more prevalent than the absence of fighting. The opposite occurred for positive descriptors such as “calm” (Figure 3), “content” (Figure 4), “relaxed” (Figure 10), and “playful” (Figure 15). Moreover, “calm” decreases slightly as the behavior of mounting the backs of other animals increases for both fighting categories. The same tendency occurred for the descriptors “content” and “relaxed”. The presence of fighting (score 1) is more prevalent in groups with higher playful behavior, when chewing without a substrate is scored as zero (Figure 15). The level of group agitation increases (*W* = 6.69; *p* < 0.001) as the behavior of mounting the backs of other animals increases for both fighting categories (Figure 2). However, the increase is more pronounced in the presence of fighting compared to the absence of fighting. A similar pattern (*p* < 0.001) was observed for the descriptors “tense” (Figure 4), “distressed” (Figure 6), “frustrated” (Figure 7), and “irritable” (Figure 8). Regarding chewing without a substrate, a similar pattern was observed for the descriptors “bored” and “frustrated”. The batches exhibited higher levels of this behavior when fights occurred in the group. Moreover, when fighting and chewing without a substrate were present, there was an increase in their boredom and frustration. In the cases of the groups of pigs being frustrated or bored, the behavior of mounting the back of another animal showed a strong association (*p* < 0.001) regardless of the presence or absence of fights.

**Figure 16:**
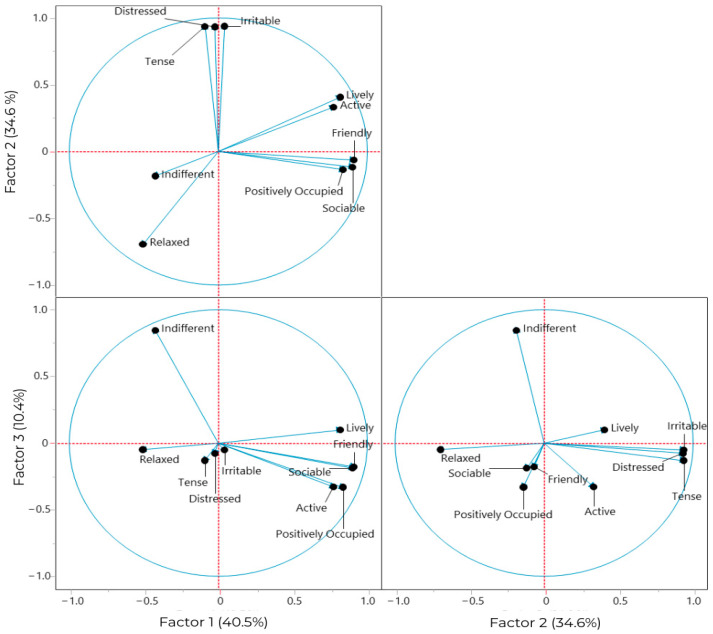
Distribution of the descriptors “active”, “distressed”, “friendly”, “indifferent”, “irritable”, “lively”, “positively occupied”, “relaxed”, “sociable”, and “tense” across the principal components (PCA factors).

The principal component analysis (PCA) of the QBA assessments for the 93 batches evaluated identified three main factors with eigenvalues greater than 1 (Table 6, Figure 16). The first factor explains 40.5% of the variance, the second factor explains 34.6%, and the third explains 10.4% of the variance. The three principal components (PCs) explain a very good proportion of the variance (85.5%). The final analysis was executed, and the factor loadings of the variables after varimax normalized rotation and the commonalities of the PCs are presented in Table 6.

Table 6 summarizes the PCA or factor analysis of various descriptors of the QBA. For instance, “sociable” has a high loading on PC1 (0.900) but nearly none on PC2 and PC3 (−0.117; −0.188), meaning it aligns strongly with PC1. “Lively” has a high loading on PC1 (0.817) and a moderate loading on PC2 (0.407). Some other variables like “active”, “friendly”, and “positively occupied” have higher loadings on PC1. The variables “relaxed” and “indifferent” contribute differently to both FLs, presenting a moderate negative association with PC1, implying that highly relaxed groups may not engage as much in social, friendly, and lively activities. However, in PC2, relaxed behavior (−0.696) is shown in the opposite plane to states of irritability, or distress, both presenting FLs higher than 0.900. These findings suggest a strong, interpretable two-factor structure underlying the behaviors, potentially distinguishing dimensions like “sociable”, “active”, “friendly”, and “positively occupied” as being highly related according to PC1 and “tense”, “distressed”, and “irritable” as being strongly related according to PC2.

Figure 16 shows the distribution of the descriptors along the three PCA factors.

Many of the descriptors exhibit strong loadings on the first principal component (PC1), which explains 40.15% of the total variance. In PC2, a close relationship is evident between the descriptors “tense”, “distressed”, and “irritable”, while “relaxed” and “indifferent” are positioned on the opposite plane. In PC1, descriptors such as “lively”, “active”, “positively occupied”, and “sociable” show a strong association with very high FLs, whereas “irritable”, “distressed”, and “tense” are also related but with very low FLs. The descriptor “indifferent”, with a high factor loading on PC3 (0.846), indicates that this adjective is strongly associated with PC3.

## 4. Discussion

A total of 93 batches of pigs, comprising 19,582 animals, were assessed in lairage pens to evaluate positive and negative descriptors of emotional states using QBA. Social behaviors and physical conditions, including signs of pathologies, were also assessed. Aggressive behaviors (including fighting, ear biting, tail biting, and mounting the backs of other animals) were observed in some batches.

Groups of pigs that were playing (*p* < 0.01) and/or were positively occupied (*p* < 0.01) displayed a positive correlation with tail manipulation. This finding may be the result of natural social interaction processes between pigs rather than being the result of stressful situations. Some studies, though, do make a clear distinction between the gentler manipulation of the tail (usually called ”tail-in-mouth behavior”) [21]. As referred to by other authors [22], playing behavior may include aggressive elements, such as head knocks and circling while maintaining shoulder-to-shoulder contact. The descriptor “playful” was negatively correlated with chewing without a substrate (*p* < 0.01). Play is a highly motivating behavior and is recognized as promoting positive animal welfare by providing environmental enrichment [23]. So, the reduction of negative behaviors such as excessive chewing is also expected. Moreover, the descriptor “playful” was negatively associated with the behavior of chewing without a substrate, indicating that higher levels of playfulness were linked to reduced chewing (Table 5). The level of playful behavior is higher when chewing without a substrate scores zero (Figure 15).

Ear biting or manipulation was positively correlated (*p* < 0.01) with the descriptor “bored”, suggesting a potential relationship between this behavior and a lack of environmental stimulation. This finding is consistent with the fact that pigs in lairage pens present difficulties in coping with the environment or performing natural behaviors [24]. Ear biting and tail biting are recognized as abnormal behaviors associated with environmental deficiencies, including poor temperature control, inadequate ventilation, high noise levels, excessive stocking densities, suboptimal diets, disease, and overall poor welfare [25,26]. The lack of chewing material, such as straw, during fasting deprives pigs of the manipulable qualities needed for investigatory behavior [27,28]. Studies show that ingestible, odorous, chewable, destructible, and manipulable deformable materials and those containing sparsely distributed edible parts are particularly appealing to pigs [28,29].

Batches experiencing higher levels of frustration or boredom showed a significant association (*p* < 0.001) with chewing behavior in the absence of a substrate. Additionally, frustration was significantly associated with the occurrence of agonistic behaviors, such as fighting (*p* = 0.004). Pigs housed in less stimulating environments are more likely to engage in manipulative social behaviors, including biting, nudging, and chewing on other pigs, as a response to boredom and the lack of appropriate outlets for natural exploratory instincts. In contrast, pigs provided with enriched environments, such as those including straw or toys, are more likely to interact with their environmental challenges rather than redirect these behaviors toward other pigs [30].

Fighting was strongly positively correlated (*p* < 0.001) with descriptors such as “agitated”, “tense”, “distressed”, “irritable”, and “frustrated”, suggesting that groups of pigs exhibiting aggressive behaviors were likely experiencing negative emotional states at the time [18]. Moreover, the descriptors “calm”, “content”, and “relaxed” were negatively associated with the occurrence of fighting, with lower scores corresponding to higher levels of fighting (Table 5). This suggests that when fighting occurs, these more positive emotional states are less prevalent. The presence of negative social behaviors, such as fighting, can serve as an indicator of these emotional disturbances [31]. Furthermore, aggression levels may vary significantly between different groups of pigs, as evidenced by the findings of Desire et al. [32], highlighting the complexity and variability of aggressive behaviors in swine populations. Consistent with [33], straw increased pig activity in pens but did not reduce fighting among newly mixed growing pigs, with aggression levels rising as the number of unfamiliar pigs increased.

Mounting behavior is a normal part of pigs’ social interactions, exhibited by both sexes, although higher incidences can be observed in males compared to females [34,35]. However, the correlations indicate that mounting the backs of other animals showed significant positive correlations (*p* < 0.001) with agitation, tension, distress, and irritability. Furthermore, the descriptors “calm” and “relaxed” were found to have significant negative associations with mounting behavior. Specifically, lower scores on these descriptors, indicating states of lesser calmness and relaxation, were strongly associated with an increase in mounting behavior. Overcrowded holding pens may promote this behavior, where one pig mounts another using its front limbs or sternum [36], which can result in visible scratches or lesions on the hindquarters of the carcass after slaughter [36].

Regarding the CPA analysis, PC1 captured a primary dimension of emotional valence in groups of pigs, with terms such as “active”, “friendly”, “positively occupied”, “sociable”, and “lively” showing strong loadings. These descriptors are associated with positive emotional states, indicating that PC1 plays a significant role in representing pigs’ overall affective welfare. A previous study on sows revealed that many descriptors also loaded strongly on PC1, ranging from “enjoying/relaxed” to “tense/frustrated”, suggesting that this component primarily reflected the emotional valence of sows’ affective states [15]. Furthermore, a similar study conducted on donkeys concluded that individuals with high positive scores on this component exhibited a more positive emotional state compared to those with high negative scores [37]. In the present study, PC2 revealed an axis of emotional contrast, with descriptors representing relaxation positioned opposite terms such as “irritable”, “distressed”, and “tense”. This suggests that PC2 could be useful for identifying conditions under which pigs experience high levels of stress. The negative relationship between relaxation and PC2 suggests that management could promote more relaxed and socially engaged behaviors. These findings emphasize the importance of PC1 and PC2 in capturing the range of emotional and behavioral states in groups of pigs, providing valuable insights into their welfare and affective experiences.

In the present study, QBA relies on subjective human assessment, which may be influenced by observer bias, potentially affecting result accuracy. The study employs behavioral assessments and physical condition observations to infer emotional states without incorporating physiological stress markers.

## 5. Conclusions

Qualitative behavior assessment (QBA) is a valuable method in animal welfare science. This study employed it alongside measures of social interaction behaviors and physical examinations to gain a comprehensive understanding of how animals perceive and respond to their environment in lairage pens. Higher levels of frustration were linked to increased occurrences of mounting and fighting behaviors. In contrast, when groups exhibited higher levels of calmness and relaxation, negative social behaviors, such as aggression, were significantly reduced. These findings highlight the importance of addressing specific welfare concerns, suggesting that targeted strategies designed to promote relaxation and reduce frustration could help mitigate negative behaviors and improve overall animal welfare in lairage pens.

## Figures and Tables

**Table 1:** Definition of the QBA descriptors (adapted from [15]).

Descriptor	Definition
Active	Not resting or sleeping and can be performing any type of motor activity.
Agitated	Display a lot of movement or is in a hurry to carry out an action or change actions.
Apathetic	Lacking in energy, vigor, and enthusiasm. Despondent and passive, not engaging with any particular element in their surroundings.
Bored	Unstimulated and uninterested.
Calm	Quiet, placid, and without strong emotions.
Content	Signs of overall satisfaction, peace, and comfort.
Distressed	Actively attempting to escape, avoid stressors, or withdraw completely; potentially experiencing panic, anxiety, or pain.
Sociable	Enjoying what they are doing, and interacting with environmental resources and their surroundings.
Friendly	Calm approaches and gentle social interactions in their environment.
Fearful	Startled, afraid, or terrified of a situation and may freeze or actively try to escape or retreat.
Frustrated	Discouraged due to repeated unsuccessful attempts to achieve or change something, hindered from reaching a goal, or consistently failing a task.
Happy	Joyful, cheerful, and untroubled.
Indifferent	Lacking interest in their environment and showing little to no attention or reaction to stimuli.
Inquisitive	Showing curiosity by exploring their surroundings, approaching objects, and investigating everything around.
Irritable	Easily reactive in a negative way towards any stimulus; uneasy and may be quick to flinch or show signs of annoyance.
Lively	Energetic, full of vigor, observant of their surroundings, and may be quick to move.
Playful	Fun-loving and gleeful. They may run, hop, spin, or manipulate items.
Positively occupied	Engaged in an activity they enjoy or require; fully immersed in their surroundings with focused, purposeful, and constructive behaviour.
Relaxed	At ease with their surroundings.
Tense	Alert and uneasy, with a worried or cautious expression, and a stiff posture.

**Table 2:** Social behaviors observed in lairage pens.

Behavior Parameters	Observed Behaviors
Aggression	Two or more pigs are fighting; biting tails; biting ears.
Tail or ear manipulation	A pig is touching another pig’s tail or ear with its snout.
Mounting	Climbing onto another animal’s back.
Exploratory behavior in pens	Exploring the floor of the pen with the snout.
Exploratory behavior in the environment	Catching water in the air with the mouth.
Exploratory behavior with enrichment materials	Environmental interaction with suspended balls.
Stereotypes	Chewing/masticating without substrate; biting bars.

**Table 3:** Spearman correlations between positive emotions (QBA descriptors) and behaviors observed in lairage pens.

	Emotions (+)	Active	Calm	Content	Happy	Lively	Inquisitive	Sociable	Friendly	Playful	Posit Occupied	Relaxed
Behavior												
Fighting		0.215	−0.305 **	−0.303 **	−0.148	0.169	0.176	0.017	−0.004	0.017	0.083	−0.288 **
Ear biting		−0.021	0.162	−0.078	0.112	−0.205	−0.057	−0.053	−0.007	0.014	0.110	0.053
Tail biting		0.262	0.058	−0.336 **	0.162	0.030	0.102	0.133	0.133	0.288 **	0.296 **	−0.165
Mounting on the back of other animals		0.225	−0.358 ***	−0.233	−0.011	0.071	0.093	0.042	0.000	0.007	0.047	−0.382 ***
Chewing without a substrate		−0.193	0.155	0.048	−0.210	−0.281	−0.116	−0.229	−0.247	−0.286 **	−0.146	0.113

** *p* < 0.01, *** *p* < 0.001.

**Table 4:** Spearman correlations between negative emotions (QBA descriptors) and behaviors observed in lairage pens.

	Emotions (−)	Agitated	Apathetic	Bored	Tense	Distressed	Fearful	Frustrated	Indifferent	Irritable
Behavior										
Fighting		0.453 ***	0.121	0.178	0.412 ***	0.432 ***	0.064	0.300 **	−0.221	0.426 ***
Ear biting		−0.010	−0.072	0.295 **	−0.088	−0.189	−0.204	0.189	−0.064	−0.074
Tail biting		0.189	−0.053	0.131	0.007	0.001	−0.129	0.105	−0.182	−0.038
Mounting on the back of other animals		0.493 ***	−0.027	0.201	0.601 ***	0.494 ***	0.197	0.385 ***	0.191	0.567 ***
Chewing without substrate		−0.086	−0.205	0.408 ***	−0.012	−0.128	−0.066	0.401 ***	−0.048	−0.128

** *p* < 0.01, *** *p* < 0.001.

**Table 5:** Results of the Kruskal-Wallis test on the association of different emotional states on social behaviors (e.g., fighting, mounting another animal’s back, chewing without substrate).

	Fighting		Score 0 vs. 1	Mounting the Backs of Other Animals		Score 0 vs. 1	Chewing Without a Substrate		Score 0 vs. 1
Emotional States	*χ* ^2^	*p*	*W*	*χ* ^2^	*p*	*W*	*χ* ^2^	*p*	*W*
Active	4.07	0.044	2.85	5.58	0.018	3.34	2.86	0.091	-
Agitated	18.88	<0.001	6.14	22.40	<0.001	6.69	0.68	0.408	-
Apathetic	1.36	0.244	-	0.07	0.798	-	3.87	0.049	−2.78
Bored	2.91	0.088	-	3.71	0.054	-	15.29	<0.001	5.53
Calm	12.29	<0.001	−4.96	21.24	<0.001	−6.52	1.14	0.285	-
Content	8.78	0.003	−4.19	4.39	0.036	−2.96	0.38	0.538	-
Distressed	17.14	<0.001	5.85	22.50	<0.001	6.71	1.50	0.221	-
Fearful	0.13	0.716	-	2.89	0.089	-	0.10	0.756	-
Friendly	0.19	0.661	-	0.17	0.682	-	3.73	0.053	-
Frustrated	8.26	0.004	4.06	13.62	<0.001	5.22	14.81	<0.001	5.44
Happy	0.39	0.534	-	0.23	0.635	-	3.20	0.074	-
Indifferent	4.49	0.034	−3.00	3.36	0.067	-	0.21	0.646	-
Inquisitive	2.84	0.092	-	0.79	0.375	-	1.24	0.265	-
Irritable	16.68	<0.001	5.78	29.58	<0.001	7.69	1.51	0.220	-
Lively	4.15	0.042	2.88	4.94	0.026	3.14	6.55	0.011	−3.62
Playful	0.03	0.871	-	0.00	0.947	-	7.51	0.006	−3.87
Positively occupied	0.64	0.424	-	0.20	0.653	-	1.95	0.162	-
Relaxed	7.64	0.006	−3.91	13.43	<0.001	−5.18	1.17	0.280	-
Sociable	0.43	0.514	-	0.75	0.385	-	3.32	0.069	-
Tense	15.64	<0.001	5.59	33.27	<0.001	8.16	0.00	0.911	-

Significant results at *p* < 0.05.

**Table 6:** Factor loadings and commonalities of the first three PCs after varimax normalized rotation.

Variable (Descriptors)	Factor Loadings (FLs)			CM ^b^
	PC1 ^a^	PC2	PC3	
Bartlett’s test of sphericity	<0.0001			
KMO ^c^ measure	0.870			
Active	0.772	0.332	−0.328	0.81
Distressed	−0.045	0.926	−0.068	0.85
Tense	−0.091	0.937	−0.130	0.88
Friendly	0.909	−0.065	−0.178	0.90
Indifferent	−0.424	−0.184	0.846	0.95
Irritable	0.041	0.939	−0.052	0.89
Lively	0.817	0.407	0.099	0.90
Positively occupied	0.838	−0.137	−0.331	0.83
Relaxed	−0.506	−0.696	−0.050	0.74
Sociable	0.900	−0.117	−0.188	0.88
Explained variance (%)	40.5	34.6	10.4	Σ = 85.5

^a^ PC—principal component; ^b^ CM—commonality; ^c^ KMO—Kaiser–Meyer–Olkin.

## Data Availability

The data can be provided upon reasonable request.

## References

[1] Saraiva S., Santos S., García-Díez J., Simões J., Saraiva C. (2024). Comparative analysis of animal welfare in three broiler slaughterhouses and associated farms with unsatisfactory slaughterhouse results. Animals.

[2] Witt J., Krieter J., Büttner K., Wilder T., Hasler M., Bussemas R., Witten S., Czycholl I. (2024). Relationship between animal-based on-farm indicators and meat inspection data in pigs. Porc. Health Manag..

[3] Čobanović N., Čalović S., Suvajdžić B., Grković N., Stanković S.D., Radaković M., Spariosu K., Karabasil N. (2024). Consequences of Transport Conditions on the Welfare of Slaughter Pigs with Different Health Status and RYR-1 Genotype. Animals.

[4] Welfare Quality® Consortium (2009). Welfare Quality® Assessment Protocol for Pigs (Sows and Piglets, Growing and Finishing Pigs).

[5] Rey-Salgueiro L., Martinez-Carballo E., Fajardo P., Chapela M.J., Espiñeira M., Simal-Gandara J. (2018). Meat quality about swine well-being after transport and during lairage at slaughterhouse. Meat Sci..

[6] Gade B.P. (2008). Effect of rearing system and mixing at loading on transport and lairage behavior and meat quality: Comparison of outdoor and conventionally raised pigs. Animal.

[7] Weeks C.A. (2008). A review of welfare in cattle, sheep, and pig lairages, with emphasis on stocking rates, ventilation, and noise. Anim. Welf..

[8] D’Eath R.B., Turner S.P., Kurt E., Evans G., Thölking L., Looft H., Wimmers K., Murani E., Klont R., Foury A. (2010). Pigs’ aggressive temperament affects pre-slaughter mixing aggression, stress, and meat quality. Animal.

[9] Geverink N.A., Engel B., Lambooij E., Wiegant V.M. (1996). Observations on behavior and skin damage of slaughter pigs and treatment during lairage. Appl. Anim. Behav. Sci..

[10] Vermeulen L., Van de Perre V., Permentier L., De Bie S., Verbeke G., Geers R. (2015). Pre-slaughter handling and pork quality. Meat Sci..

[11] Faucitano L. (2018). Preslaughter handling practices and their effects on animal welfare and pork quality. J. Anim. Sci..

[12] Camerlink I., Peijnenburg M., Wemelsfelder F., Turner S.P. (2016). Emotions after victory or defeat assessed through qualitative behavioral assessment, skin lesions, and blood parameters in pigs. Appl. Anim. Behav. Sci..

[13] Geverink N.A., Bühnemann A., van de Burgwal J.A., Lambooij E., Blokhuis H.J., Wiegant V.M. (1998). Responses of slaughter pigs to transport and lairage sounds. Physiol. Behav..

[14] Giuliotti L., Benvenuti M.N., Giannarelli A., Mariti C., Gazzano A. (2019). Effect of different environment enrichments on behavior and social interactions in growing pigs. Animals.

[15] Ibach S., Chou J.-Y., Battini M., Parsons T.D. (2024). A systematic approach to defining and verifying descriptors used in the Qualitative Behavioral Assessment of sows. Anim. Welf..

[16] Yeates J.W., Main D.C.J. (2008). Assessment of Positive Welfare: A Review. Vet. J..

[17] Boissy A., Manteuffel G., Jensen M.B., Moe R.O., Spruijt B., Keeling L.J., Winckler C., Forkman B., Dimitrov I., Langbein J. (2007). Assessment of positive emotions in animals to improve their welfare. Physiol. Behav..

[18] Temple D., Manteca X., Velardo A., Almau A. (2011). Assessment of Animal Welfare through Behavioural Parameters in Iberian Pigs in Intensive and Extensive Conditions. Appl. Anim. Behav. Sci..

[19] Schmitt O., O’Driscoll K., Baxter E., Boyle L. (2019). Artificial Rearing Affects the Emotional State and Reactivity of Pigs Post-Weaning. Anim. Welf..

[20] Fleming P.A., Clarke T., Wickham S.L., Stockman C.A., Barnes A.L., Collins T., Miller D.W. (2016). The Contribution of Qualitative Behavioural Assessment to Appraisal of Livestock Welfare. Anim. Prod. Sci..

[21] Brunberg E., Wallenbeck A., Keeling L.J. (2011). Tail biting in fattening pigs: Associations between frequency of tail biting and other abnormal behaviors. Appl. Anim. Behav. Sci..

[22] Newberry R.C., Wood-Gush D.G.M., Hall J.W. (1988). Playful behavior of piglets. Behav. Process..

[23] Steinerová K., Parker S.E., Brown J.A., Seddon Y.M. (2024). The Promotion of Play Behaviour in Grow-Finish Pigs: The Relationship between Behaviours Indicating Positive Experience and Physiological Measures. Appl. Anim. Behav. Sci..

[24] Diana A., Carpentier L., Piette D., Boyle L.A., Berckmans D., Norton T. (2019). An ethogram of biter and bitten pigs during an ear-biting event: First step in the development of a precision livestock farming tool. Appl. Anim. Behav. Sci..

[25] Henry M., Jansen H., Amezcua M.D., O’Sullivan T.L., Niel L., Shoveller A.K., Friendship R.M. (2021). Tail-biting in pigs: A scoping review. Animals.

[26] Boyle L.A., Edwards S.A., Bolhuis J.E., Pol F., Šemrov M.Z., Schütze S., Nordgreen J., Bozakova N., Sossidou E.N., Valros A. (2022). The evidence for a causal link between disease and damaging behavior in pigs. Front. Vet. Sci..

[27] Telkänranta H., Swan K., Hirvonen H., Valros A. (2014). Chewable materials before weaning reduce tail biting in growing pigs. Appl. Anim. Behav. Sci..

[28] Jensen M.B., Herskin M.S., Forkman B., Pedersen L.J. (2015). Effect of Increasing Amounts of Straw on Pigs’ Explorative Behaviour. Appl. Anim. Behav. Sci..

[29] Studnitz M., Jensen M.B., Pedersen L.J. (2007). Why Do Pigs Root and in What Will They Root? A Review on the Exploratory Behaviour of Pigs in Relation to Environmental Enrichment. Appl. Anim. Behav. Sci..

[30] De Jong I.C., Ekkel E.D., Van de Burgwal J.A., Lambooij E., Korte S.M., Ruis M.A.W., Koolhaas J.M., Blokhuis H.J. (1998). Effects of straw bedding on physiological responses to stressors and behavior in growing pigs. Physiol. Behav..

[31] Dalmau A., Velarde A., Velarde A., Raj M. (2016). Lairage and handling. Animal Welfare at Slaughter.

[32] Desire S., Turner S.P., D’Eath R.B., Doeschl-Wilson A.B., Lewis C.R.G., Roehe R. (2015). Analysis of the phenotypic link between behavioral traits at mixing and increased long-term social stability in group-housed pigs. Appl. Anim. Behav. Sci..

[33] Arey D.S., Franklin M.F. (1995). Effects of Straw and Unfamiliarity on Fighting Between Newly Mixed Growing Pigs. Appl. Anim. Behav. Sci..

[34] Rydhmer L., Zamaratskaia G., Andersson H.K., Algers B., Guillemet R., Lundström K. (2006). Aggressive and sexual behavior of growing and finishing pigs reared in groups, without castration. Acta Agric. Scand. Sect. A—Anim. Sci..

[35] Hemsworth P.H., Tilbrook A.J. (2007). Sexual behavior of male pigs. Horm. Behav..

[36] Driessen B., Van Beirendonck S., Buyse J. (2020). Effects of transport and lairage on the skin damage of pig carcasses. Animals.

[37] Minero M., Dalla Costa E., Dai F., Murray L.A., Canali E., Wemelsfelder F. (2016). Use of Qualitative Behavior Assessment as an indicator of welfare in donkeys. Appl. Anim. Behav. Sci..

